# Increased Mortality from Extrapancreatic Infections in Hospitalized Patients with Acute Pancreatitis

**DOI:** 10.1155/2019/2789764

**Published:** 2019-02-28

**Authors:** Guido Grajales-Figueroa, Héctor Adrián Díaz Hernández, Martín Alejandro Chacón Portillo, Luis F. Uscanga, Mario Peláez-Luna, Jorge Hernández Calleros

**Affiliations:** ^1^National Institute of Medical Science and Nutrition Salvador Zubiran, Department of Endoscopy, Mexico City, Mexico; ^2^National Institute of Medical Science and Nutrition Salvador Zubiran, Department of Gastroenterology, Mexico City, Mexico; ^3^Division of Congenital Heart Surgery, Texas Children's Hospital, Michael E. DeBakey Department of Surgery, Baylor College of Medicine, Houston, Texas, USA

## Abstract

Nosocomial extrapancreatic infections in patients with acute pancreatitis (AP) are associated with a higher mortality even after adjusting the risk for the severity of the pancreatitis. The aim of this study was to describe the clinical features of hospitalized patients with AP who died during their hospitalization and to evaluate risk factors associated with mortality. We performed a descriptive study of the clinical features of adult patients who died from AP during their hospitalization and a case control study with a paired group of patients that survived AP during a 10-year period. Data of interest were collected from the medical records and are presented with appropriate measures of central tendency and dispersion. For the case control study, the primary outcome evaluated was death, and to evaluate associated clinical features and determine differences between groups, we performed the *χ*^2^ or Fisher's exact tests for categorical variables and the Student *t*-test or Mann-Whitney *U* test for continuous variables as appropriate. We found 48 patients with acute pancreatitis who died within the period of the study during hospitalization; from these, 50% were men, mean age was 53.2 years, and the most common etiology was biliary obstruction by gallstones in 45.8%. The global mortality rate was of 2.5%. A total of 43.7% patients had infected pancreatic necrosis, and in 58.3%, some extrapancreatic infection was documented, being the most common urinary tract infection in 50%, bacteremia in 50% and pneumonia in 33.3%. Clinical features associated with mortality were the presence of organ failure (*p* < 0.001), nosocomial complications (*p* < 0.001), infected necrosis (*p* < 0.001), and extrapancreatic infections (*p* = 0.002). From the different extrapancreatic infections, only bacteremia (*p* = 0.001) and pneumonia (*p* = 0.011) were associated with higher mortality. In conclusion, extrapancreatic infections are associated with increased mortality among hospitalized patients with acute pancreatitis, in particular, bacteremia and pneumonia with an isolated pathogen.

## 1. Introduction

Acute pancreatitis (AP) is an acute inflammatory process of the pancreas clinically characterized by abdominal pain and elevated pancreatic enzymes [1]. It is a common pathology of global distribution that can occasionally be life threatening. Global estimates of incidence range from 33 to 74 cases per 100,000 person-years and it represents the third most common cause of gastrointestinal (GI) disease hospital admissions in developed countries [2, 3]. AP accounts for 1-60 deaths per 100,000 person-years worldwide, with an estimated mortality rate of 2-9% according to different series [2, 4]. The diagnosis of acute pancreatitis requires the presence of two criteria from characteristic abdominal pain, elevated serum levels of pancreatic enzymes, and characteristic findings in imaging studies [1]. Historically, multiple severity scores have been used for the evaluation of AP severity. Until recently, the most accurate scores to differentiate mild from severe acute pancreatitis have been the Ranson score, the acute physiology and chronic health evaluation (APACHE) II score, and the computed tomography (CT) severity index [5]. Currently, acute pancreatitis severity is classified into mild when there is no organ failure, local or systemic complication; moderately severe, when there is a transient organ failure, local or systemic complication; and severe, when there is one or more persistent organ failure. The definition of organ failure is based on the modified Marshall scoring system [1].

Patients with AP can develop pancreatic infections such as infected necrosis, pancreatic abscesses, and/or infected pseudocysts. The most commonly isolated microorganisms are gram-negative bacteria including *Escherichia coli*, *Pseudomonas aeruginosa*, and *Enterobacteriaceae* [6]. Recently, gram-positive and anaerobic bacteria have also been found to be responsible for pancreatic infections [6–8]. Regarding fungal infections, *Candida* spp. are responsible for approximately 15-30% of pancreatic infections and are associated with more severe systemic complications and decreased survival [9–11].

Nosocomial extrapancreatic infections have received more attention in the recent years, as they are associated with a higher mortality in patients with AP even after adjusting the risk for the severity of the disease, suggesting that an exhaustive evaluation of extrapancreatic infections should be considered when a patient with AP is deteriorating, and if detected, it must be treated aggressively [12–15]. In a recent study that evaluated the outcomes of abdominal surgical interventions in patients with severe acute pancreatitis and intra-abdominal hypertension, no difference was observed on the incidence of infected pancreatic necrosis or extrapancreatic infections between operated and not-operated patients, despite the fact that the operated patients had a longer intrahospital stay [16].

The main objective of this study was to describe the clinical features of hospitalized patients with AP who died during their hospitalization in the National Institute of Medical Science and Nutrition Salvador Zubiran during a period of 10 years. And secondarily, we aimed to evaluate risk factors associated with mortality in hospitalized patients with AP, comparing the case group of patients who died with a control group of patients with AP who did not die during hospitalization.

## 2. Materials and Methods

### 2.1. Study Design

We performed an observational, descriptive, retrospective study of patients who died from AP during their hospitalization, with a subsequential case control study in which a paired group of patients with AP that survived at the end of their hospitalization was used as controls.

### 2.2. Study Population

An exhaustive search was performed in the electronic records of our tertiary care center for previously hospitalized patients with the diagnosis of AP, notwithstanding the original reason for admission, during the period from July 1, 2000, to July 30, 2010. Afterward, a second search was performed for patients from both sexes of 18 years or more who have died during hospitalization. Once the patients were identified, a review of their clinical records was accomplished for data collection and analysis. For the control population, we included one control per case paired by sex, age, pancreatitis etiology, and comorbidities, who have had an episode of AP during the same period of time, but whose outcome of hospitalization would have been discharge for improvement or cure. Finally, a case control study was performed to identify risk factors associated with mortality. We excluded patients whom medical record was incomplete for the data required for the analysis.

### 2.3. Data Collection

The following variables were collected from medical records: sex, age, etiology of AP, Ranson score, APACHE II score, comorbidities, organ failures, requirement of renal substitution therapy, mechanical ventilation or vasopressors, presence of infected necrosis and isolated microorganisms, presence of extrapancreatic infections and isolated microorganisms (respiratory, urinary tract, bacteremia, and catheter-associated), nosocomial-associated complications, requirement of surgical management, and length of hospitalization.

Severe AP was defined by the Ranson score (≥3) and the APACHE II score (≥8); lower scores were categorized as mild AP. Organ failures were defined by the modified Marshal scoring system for organ dysfunction proposed in the Atlanta international consensus. Organ failure was defined as a score of two or more in any respiratory, renal, or cardiovascular system. Respiratory failure was defined as PaO2/FiO2 ≤ 300, renal failure was defined as serum creatinine > 1.8 mg/dL, and cardiovascular failure was defined as systolic blood pressure < 90 mmHg, not fluid responsive.

### 2.4. Statistical Analysis

For the descriptive analyses, categorical variables are presented as number and percentage, continuous normally distributed variables are presented as mean (m) and standard deviation (SD), and continuous nonnormally distributed variables are presented as median (M) and interquartile range (IQR). For the case control study, the primary outcome evaluated was death. To determine differences between groups, categorical variables were analyzed using *χ*^2^ or Fisher's exact tests depending on sample size, and continuous variables were analyzed using Student's *t*-test for normally distributed variables and Mann-Whitney *U* test for nonnormally distributed variables. Statistical significance was established with a *p* value < 0.05. All analyses were performed using Stata statistical package version 10.1. A biomedical statistician performed the statistical review.

## 3. Results

**Figure 1:**
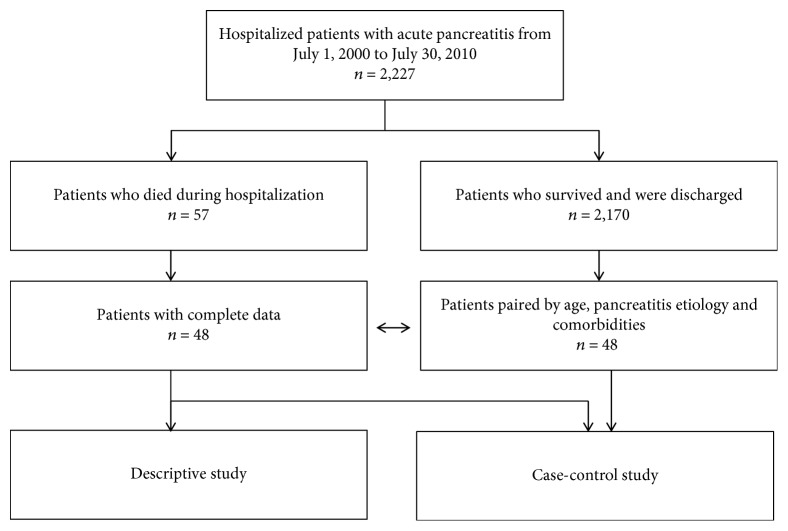
Flow diagram of the patients included in the study.

Over the study period, a total of 2227 patients with AP were hospitalized in our tertiary care center. Of these, 57 patients died during hospitalization, representing a global mortality rate of 2.5%. Complete data were available only from 48 medical records. A flow diagram is shown in Figure 1.

From these, 24 (50%) were men, mean age was 53.2 (SD ± 18.2) years, and the most common etiology was mechanical biliary tract obstruction by gallstones in 22 (45.8%) of patients. Complete demographics are shown in Table 1.

With regard to the severity of the AP episodes, Ranson score was applied to categorize 37 patients, of whom 26 (70.2%) qualified as severe AP. APACHE II score was calculated in 25 patients, of whom 19 (76%) qualified as severe AP. Taking the occurrence of organ failure (OF) as a severity index, 46 (95.8%) patients presented at least one OF.  Table 2 shows the Ranson scores, APACHE II scores, and OF distribution among the patients with AP who died during hospitalization.

A total of 21 (43.7%) patients had infected pancreatic necrosis. Of these cases, 16 (76.7%) were polymicrobial infections. The most commonly isolated microorganisms were *Staphylococcus* spp. (47.6%), *Enterococcus faecium* (42.8%), and *Pseudomonas aeruginosa* (33.3%).  Table 3 shows the isolated microorganisms from infected pancreatic necrosis.

On the other hand, 28 (58.3%) patients presented extrapancreatic infections; of these, 16 (57.1%) presented more than one different extrapancreatic infection. A total of 54 extrapancreatic infections were identified; of which, 19 (35%) were polymicrobial. From the patients with extrapancreatic infections, 14 (50%) presented bacteremia, 14 (50%) presented urinary tract infections, 12 (42.8%) presented pneumonia, 2 (7.1%) presented catheter-related infections, and 12 (42.8%) presented some different extrapancreatic infection.  Table 4 shows the isolated microorganisms from extrapancreatic infections. A total of 27 (56.2%) patients presented nosocomial complications. The most common complications were acute respiratory failure in 2 (7.4%) patients and acute tubular necrosis in another 2 (7.4%) patients. Only one patient presented a pancreatic pseudocyst. Surgical procedures were performed in 28 (58.3%) patients. The most common procedures were necrosectomy in 20 (71.4%) patients and laparotomy in 6 (21.4%) patients.

When comparing both groups, deceased and not, no difference was found regarding the APACHE II scoring system. Statistically significant differences were established between patients with OF (*p* < 0.001), nosocomial complications (*p* < 0.001), infected necrosis (*p* < 0.001), and extrapancreatic infections (*p* = 0.002). Also, after infection site analysis, a statistically significant difference was shown for bacteremia (*p* = 0.001) and pneumonia with positive sputum culture (*p* = 0.011). All these analyses are shown in Table 5. Additionally, in a secondary analysis in which we included the 96 patients (cases and controls), but separated depending on whether or not they had extra-pancreatic infections, these last were associated with the presence of infected pancreatic necrosis and OF as shown in Table 6.

## 4. Discussion

In the present study, we found an overall in-hospital mortality of 2.5% in patients with AP, a lower rate than those reported in other centers [17–19]. This difference could be due to the fact that our center has an experienced multidisciplinary team for the management of pancreatic diseases, being a reference tertiary care hospital. These data will prove very useful in our country given the lack of reports on the characteristics of patients with acute pancreatitis in our population.

We confirm previously described associations between severity scoring systems, organ failures, and infected pancreatic necrosis with mortality in patients with AP. Nevertheless, in our study, we report the microbiological isolates of infected pancreatic tissue from the patients who died of infected pancreatic necrosis. These results support previous reports concerning the colonization of infected pancreatic necrosis by multiple microorganisms, both gram-negative and gram-positive bacteria, as well as *Candida* spp. Differing from previous findings in other centers, the most common microorganism isolated in our population was *Staphylococcus* spp. rather than *E. coli*. This supports the results of more recent studies and may suggest an epidemiological transition in infected pancreatic necrosis, which is concerning as *Staphylococcus* spp. is characterized by its increasing resistance to antibiotics. Also, prolonged hospitalizations, use of intravascular catheters, and presence of bacteremia have been identified as predisposing factors for *Staphylococcus* spp. colonization of necrotic pancreatic tissue in these patients [7, 8].

The nosocomial extrapancreatic infections have not been the focus of attention of researchers until recent years. In a retrospective Dutch study of 731 patients with AP, 26% developed extrapancreatic infections including pneumonia and/or bacteremia. The presence of an extrapancreatic infection was found to be a risk factor for developing infected necrosis, and in 61% of pancreatic infection cases, the same pathogen was isolated from extrapancreatic samples [12]. In a prospective Spanish study of 176 patients with AP, 25% developed extrapancreatic infections including pneumonia, urinary tract infections, catheter line infections, and bacteremia. The presence of pneumonia and/or bacteremia was associated with increased mortality [13]. In an American study of 11,046 patients with AP, 15% of the causes of death were attributed to a nosocomial infection [14]. Extrapancreatic infections are associated with a higher mortality in patients with AP even after adjusting the risk for the severity of the disease [15]. In our study, we found that extrapancreatic infections are associated with a higher mortality, in particular, bloodstream and lower respiratory tract infections documented with positive blood and sputum cultures, respectively, which agrees with the findings documented in recent previous studies. Furthermore, patients with extrapancreatic infections presented a higher rate of infected pancreatic necrosis and organ failure with a statistically significant difference. No further differences were found among patients with extrapancreatic infections regarding comorbidities and other nosocomial complications.

As a result of this study's limitations, such as its retrospective nature, the small sample size, and the lack of analysis of the different treatments instituted for the management of acute pancreatitis and its complications and other covariates of known relevance in AP patients' mortality, further research is required in our country with a larger sample size and prospective design in order to support these results. Regarding the generalizability of our results, these have internal validity in our country since our hospital is a reference center for pancreatic diseases; however, our results cannot be assumed to have external validity to other countries.

## 5. Conclusion

In conclusion, this study supports the fact that extrapancreatic infections are associated with increased mortality among hospitalized patients with acute pancreatitis, in particular, bacteremia and pneumonia with positive cultures.

## Figures and Tables

**Table 1:** Demographic characteristics of patients with acute pancreatitis who died and who survived during hospitalization.

Values	Patients with AP who died during hospitalization (cases) *n* = 48	Patients with AP who survived hospitalization (controls) *n* = 48	*p* value
Age in years, m (SD)	53.2 (±18.2)	53.2 (±18.2)	1.000
Males, *n* (%)	24 (50)	24 (50)	1.000
Etiology			
Biliary, *n* (%)	22 (45.8)	22 (45.8)	1.000
Alcohol, *n* (%)	7 (14.5)	7 (14.5)	1.000
Triglycerides, *n* (%)	6 (12.5)	6 (12.5)	1.000
Drugs, *n* (%)	3 (6.2)	3 (6.2)	1.000
ERCP, *n* (%)	1 (2.0)	1 (2.0)	1.000
Other, *n* (%)	9 (18.7)	9 (18.7)	1.000
Comorbidities			
Diabetes mellitus, *n* (%)	7 (14.5)	7 (14.5)	1.000
Hypertension, *n* (%)	7 (14.5)	7 (14.5)	1.000
Obesity, *n* (%)	1 (2.0)	1 (2.0)	1.000
Systemic lupus, *n* (%)	6 (12.5)	6 (12.5)	1.000
Coronary artery disease, *n* (%)	3 (6.2)	3 (6.2)	1.000
Other, *n* (%)	1 (22.9)	1 (22.9)	1.000
Days of in-hospital stay, M (IQR)	15 (4.5-53.5)	5.5 (2-13.5)	<0.05

AP: acute pancreatitis; SD: standard deviation; ERCP: endoscopic retrograde cholangiopancreatography; IQR: interquartile range.

**Table 2:** Severity scores and organ failures among patients with acute pancreatitis who died during hospitalization.

Values	Patients with AP who died during hospitalization *n* = 48
*Ranson score*	
Mild acute pancreatitis, *n* (%)	11 (29.7)
Severe acute pancreatitis, *n* (%)	26 (70.2)
*APACHE II score*	
Mild acute pancreatitis, *n* (%)	6 (24)
Severe acute pancreatitis, *n* (%)	19 (76)
Organ failure, *n* (%)	46 (95.8)
Renal, *n* (%)	25 (52)
Serum creatinine (mg/dL), M (IQR)	2.1 (1.4-4.2)
Dialysis, *n* (%)	11 (22.9)
Respiratory, *n* (%)	43 (89.5)
Mechanical ventilation, *n* (%)	41 (85.4)
Cardiovascular, *n* (%)	42 (87.5)
Use of vasoactive drugs, *n* (%)	42 (87.5)

AP: acute pancreatitis; APACHE: acute physiology and chronic health evaluation; IQR: interquartile range.

**Table 3:** Microbiological isolates in infected necrosis of patients with acute pancreatitis who died during hospitalization.

Isolates in infected necrosis	Patients with AP and infected necrosis who died during hospitalization *n* = 21
*Staphylococcus* spp., *n* (%)	10 (47.6)
*Enterococcus faecium*, *n* (%)	9 (42.8)
*Pseudomonas aeruginosa*, *n* (%)	7 (33.3)
*Candida* spp., *n* (%)	6 (28.5)
*Escherichia coli*, *n* (%)	5 (23.8)

AP: acute pancreatitis.

**Table 4:** Microbiological isolates in extrapancreatic infections of patients with acute pancreatitis who died during hospitalization.

Isolates in extrapancreatic infections	Proportions from the total extrapancreatic infections presented in the 28 affected patients with AP *n* = 54
Bacteremia	*n* = 14
*Staphylococcus* spp., *n* (%)	9 (64.2)
*Candida* spp., *n* (%)	3 (21.4)
*Escherichia coli*, *n* (%)	2 (14.2)
*Enterococcus* spp., *n* (%)	2 (14.2)
*Stenotrophomonas maltophilia*, *n* (%)	1 (7.1)
*Trichosporon beigelii*, *n* (%)	1 (7.1)
Urinary tract infections	*n* = 14
*Candida* spp., *n* (%)	9 (64.2)
*Enterococcus* spp., *n* (%)	6 (42.8)
*Staphylococcus* spp., *n* (%)	1 (7.1)
*Acinetobacter calcoaceticus*, *n* (%)	1 (7.1)
*Mycobacterium bovis*, *n* (%)	1 (7.1)
Pneumonia	*n* = 12
*Pseudomonas aeruginosa*, *n* (%)	6 (50)
*Stenotrophomonas maltophilia*, *n* (%)	4 (33.3)
*Staphylococcus* spp., *n* (%)	3 (25)
*Enterococcus* spp., *n* (%)	3 (25)
*Escherichia coli*, *n* (%)	1 (8.3%)
*Acinetobacter calcoaceticus*, *n* (%)	1 (8.3%)
*Achromobacter xylosoxidans*, *n* (%)	1 (8.3%)
*Candida* spp., *n* (%)	1 (8.3%)
*Cryptococcus neoformans*, *n* (%)	1 (8.3%)
Catheter-related infections	*n* = 2
*Staphylococcus* spp., *n* (%)	1 (50)
*Pseudomonas aeruginosa*, *n* (%)	1 (50)
*Candida* spp., *n* (%)	1 (50)
Other	*n* = 12
*Enterococcus* spp., *n* (%)	6 (50)
*Escherichia coli*, *n* (%)	4 (33.3)
*Staphylococcus* spp., *n* (%)	2 (16.6)
*Pseudomonas aeruginosa*, *n* (%)	2 (16.6)
*Candida* spp., *n* (%)	2 (16.6)
*Klebsiella pneumoniae*, *n* (%)	1 (8.3)
*Pseudomonas oryzihabitans*, *n* (%)	1 (8.3)
*Clostridium difficile*, *n* (%)	1 (8.3)
*Citrobacter freundii*, *n* (%)	1 (8.3)
*Trichosporon beigelii*, *n* (%)	1 (8.3)
*Entamoeba histolytica*, *n* (%)	1 (8.3)

AP: acute pancreatitis.

**Table 5:** Characteristics of patients with acute pancreatitis associated with mortality during hospitalization.

Values	Patients with AP who died during hospitalization (cases) *n* = 48	Patients with AP who survived hospitalization (controls) *n* = 48	*p* value
Ranson score, m (SD)	3.05 (±1.45)	1.54 (±1.25)	<0.01
Organ failure, *n* (%)	46 (95.8)	10 (20.8)	<0.01
Renal failure, *n* (%)	25 (52.0)	6 (12.5)	<0.01
Serum creatinine (mg/dL), M (IQR)	2.1 (1.45-4.28)	0.8 (0.7-1.1)	<0.01
Dialysis, *n* (%)	11 (22.9)	2 (4.1)	<0.01
Respiratory failure, *n* (%)	43 (89.5)	5 (10.4)	<0.01
Mechanical ventilation, *n* (%)	41 (85.4)	4 (8.3)	<0.01
Cardiovascular failure, *n* (%)	42 (87.5)	3 (6.2)	<0.01
Use of vasoactive drugs, *n* (%)	42 (87.5)	3 (6.2)	<0.01
Infected pancreatic necrosis, *n* (%)	21 (43.7)	3 (6.2)	<0.01
Other infection, *n* (%)	28 (58.3)	13 (27.0)	<0.01
Bacteremia, *n* (%)	14 (29.1)	2 (4.1)	<0.01
Pneumonia, *n* (%)	12 (25)	3 (6.2)	<0.05
Other complication, *n* (%)	27 (56.2)	10 (20.8)	<0.01
Days of in-hospital stay, M (IQR)	15 (4.5-53.5)	5.5 (2-13.5)	<0.05

AP: acute pancreatitis; SD: standard deviation; IQR: interquartile range.

**Table 6:** Complications associated with the presence of extrapancreatic infections in 96 patients hospitalized with acute pancreatitis (cases and controls).

Values	Patients with AP and extrapancreatic infection *n* = 41	Patients with AP and without extrapancreatic infection *n* = 55	*p* value
Infected pancreatic necrosis, *n* (%)	19 (46.3)	5 (9.0)	<0.001
Renal failure, *n* (%)	18 (43.9)	13 (23.6)	<0.05
Respiratory failure, *n* (%)	31 (75.6)	17 (30.9)	<0.001
Cardiovascular failure, *n* (%)	27 (65.8)	18 (32.7)	<0.01
Comorbidities	29 (70.7)	43 (78.1)	0.171
Other complications, *n* (%)	20 (48.7)	17 (30.9)	0.075

AP: acute pancreatitis.

## Data Availability

The data from the patients comes from their clinical records of our center and that information is confidential.

## References

[1] Banks P. A., Bollen T. L., Dervenis C. (2012). Classification of acute pancreatitis—2012: revision of the Atlanta classification and definitions by international consensus. *Gut*.

[2] Xiao A. Y., Tan M. L. Y., Wu L. M. (2016). Global incidence and mortality of pancreatic diseases: a systematic review, meta-analysis, and meta-regression of population-based cohort studies. *The Lancet Gastroenterology & Hepatology*.

[3] Everhart J. E., Ruhl C. E. (2009). Burden of digestive diseases in the United States part III: liver, biliary tract, and pancreas. *Gastroenterology*.

[4] Banks P. A., Freeman M. L., the Practice Parameters Committee of the American College of Gastroenterology (2006). Practice guidelines in acute pancreatitis. *The American Journal of Gastroenterology*.

[5] Fischer A. J., Andreottola F., Lenz P., Lebiedz P. (2017). Akute pankreatitis in der intensivmedizin. *Medizinische Klinik - Intensivmedizin und Notfallmedizin*.

[6] Garg P. K., Khanna S., Bohidar N. P., Kapil A., Tandon R. K. (2001). Incidence, spectrum and antibiotic sensitivity pattern of bacterial infections among patients with acute pancreatitis. *Journal of Gastroenterology and Hepatology*.

[7] Rau B., Bothe A., Beger H. G. (2005). Surgical treatment of necrotizing pancreatitis by necrosectomy and closed lavage: changing patient characteristics and outcome in a 19-year, single-center series. *Surgery*.

[8] Sahar N., Kozarek R. A., Kanji Z. S. (2018). The microbiology of infected pancreatic necrosis in the era of minimally invasive therapy. *European Journal of Clinical Microbiology & Infectious Diseases*.

[9] Isenmann R., Schwarz M., Rau B., Trautmann M., Schober W., Beger H. G. (2002). Characteristics of infection with Candida species inpatients with necrotizing pancreatitis. *World Journal of Surgery*.

[10] Kujath P., Rosenfeldt M., Esnaashari H., Scheele J., Bouchard R. (2005). Pilzinfektion bei hamorrhagisch-nekrotisierender pankreatitis: risikofaktoren, inzidenz, therapie. Fungal infections in patients with necrotizing pancreatitis: risk-factors, incidence, therapy. *Mycoses*.

[11] Reuken P. A., Albig H., Rödel J. (2018). Fungal infections in patients with infected pancreatic necrosis and pseudocysts: risk factors and outcome. *Pancreas*.

[12] Besselink M. G., van Santvoort H. C., Boermeester M. A. (2009). Timing and impact of infections in acute pancreatitis. *British Journal of Surgery*.

[13] Pando E., Alberti P., Hidalgo J. (2018). The role of extra-pancreatic infections in the prediction of severity and local complications in acute pancreatitis. *Pancreatology*.

[14] Wu B. U., Johannes R. S., Kurtz S., Banks P. A. (2008). The impact of hospital-acquired infection on outcome in acute pancreatitis. *Gastroenterology*.

[15] Noor M. T., Radhakrishna Y., Kochhar R. (2011). Bacteriology of infection in severe acute pancreatitis. *Journal of the Pancreas: JOP*.

[16] Hernández H. A. D., Thomas J. A. G., Solís H. T., Luna M. C. P., Domínguez L. F. U., Calleros J. H. (2017). The impact of surgery on mortality and morbidity in patients with severe acute pancreatitis and intra-abdominal hypertension. *Cogent Medicine*.

[17] Cavallini G., Frulloni L., Bassi C. (2004). Prospective multicentre survey on acute pancreatitis in Italy (ProInf-AISP): results on 1005 patients. *Digestive and Liver Disease*.

[18] Fu C. Y., Yeh C. N., Hsu J. T., Jan Y. Y., Hwang T. L. (2007). Timing of mortality in severe acute pancreatitis: experience from 643 patients. *World Journal of Gastroenterology*.

[19] Schepers N. J., Bakker O. J., Besselink M. G. (2018). Impact of characteristics of organ failure and infected necrosis on mortality in necrotising pancreatitis. *Gut*.

